# LB1580. Efficacy Assessment of MAT2203 Encochleated Oral Formulation of Amphotericin B, in the Neutropenic Mouse Model of Pulmonary Mucormycosis

**DOI:** 10.1093/ofid/ofac492.1890

**Published:** 2022-12-15

**Authors:** Teclegiorgis Gebremariam, Yiyou Gu, Sondus Alkhazraji, Eman Youssef, Theresa Matkovits, Jenel Cobb, Raphael J Mannino, Ashraf S Ibrahim

**Affiliations:** The Lundquist Institute at Harbor-UCLA Medical Center, Torrance, California; The Lundquist Institute at Harbor-UCLA Medical Center, Torrance, California; The Lundquist Institute at Harbor-UCLA Medical Center, Torrance, California; The LUndquist Institute, Torrance, California; Matinas BioPharma, Bedminster, New Jersey; Matinas BioPharma, Bedminster, New Jersey; Matinas BioPharma, Bedminster, New Jersey; The Lundquist Institute at Harbor-UCLA Medical Center, Torrance, California

## Abstract

**Background:**

Invasive mucormycosis (IM) is associated with high mortality and morbidity. MAT2203 is an encochleated oral formulation of amphotericin B which has been shown to be safe and effective against murine aspergillosis and murine cryptococcal meningoencephalitis. We sought to compare the efficacy of MAT2203 to liposomal amphotericin B (LAMB) treatment in a neutropenic mouse model of IM.

**Methods:**

ICR mice were immunosuppressed with cyclophosphamide and cortisone acetate on days -2, -3 and +8, relative to infection with intratracheally instilled *Rhizopus delemar* 99-880 or *M. circinelloides f. jenssenii* DI15-131. Treatment with placebo (diluent control), oral MAT2203 (5 to 45 mg/kg, given qd or bid for 7 days) or LAMB (10 mg/kg, iv, qd for 4 days), began 16 h post infection. Survival (n=10-20/group from 1/2 experiments) through Day +21 and tissue fungal burden of lungs or brain (n=10/group) euthanized on Day +4 post infection (conidial equivalents using qPCR) served as a primary and secondary endpoint, respectively.

**Results:**

For *Rhizopus delemar infection*, doses of MAT2203 5,15 mg/kg qd or 7.5 mg/kg bid, significantly prolonged median survival time (MST) and enhanced overall survival *vs.* placebo-treated mice (MST of 9 Days and 0% survival for placebo-treated mice *vs.* 13 Days, and 20-40% for MAT2203 doses, *P*< 0.05 by Log-Rank). Importantly, MAT2203 treatments were as effective as LAMB (MST of 16 days and overall survival of 45%). For mice infected with *M. circinelloides*, MAT2203 at 15 mg/kg, qd significantly prolonged MST and enhanced overall survival *vs.* placebo-treated mice (MST of 5 Days and 0% survival, for placebo-treated mice *vs.* 13.5 Days and 50% survival for MAT2203, *P*< 0.05). In both infection models MAT2203 treatment of 15 mg/kg or LAMB resulted in significant ∼1.0-1.5 log reduction and ∼2.0-2.2 log reduction in lung and brain fungal burden *vs.* placebo, respectively (Wilcoxon Rank Sum).

**Conclusion:**

MAT2203 demonstrated *in vivo* efficacy in treating *R. delemar or M. circinelloides* pulmonary infection in immunosuppressed mice, which was equivalent to the LAMB current standard of care. Continued investigation and development of MAT2203 as a novel, and oral formulation of amphotericin antifungal agent against mucormycosis is warranted.

**Disclosures:**

**Theresa Matkovits, PhD**, Matinas BioPharma: Employee **Jenel Cobb, PhD**, Matinas BioPharma: Employee **Raphael J. Mannino, PhD**, Matinas BioPharma: Employee **Ashraf S. Ibrahim, PhD**, Matinas BioPharma: Advisor/Consultant|Matinas BioPharma: Grant/Research Support|SFunga: Grant/Research Support.

